# Leveraging AI Large Language Models for Writing Clinical Trial Proposals in Dermatology: Instrument Validation Study

**DOI:** 10.2196/76674

**Published:** 2026-01-12

**Authors:** Megan Hauptman, Daniel Copley, Kelly Young, Tran Do, Joseph S Durgin, Albert Yang, Jungsoo Chang, Allison Billi, Mio Nakamura, Trilokraj Tejasvi

**Affiliations:** 1 Department of Dermatology, University of Michigan, 1500 E Medical Center Dr, Ann Arbor, MI, 48105, United States, 1 734-936-4054; 2 Anduril, Costa Mesa, CA, United States

**Keywords:** artificial intelligence, AI, large language model, research proposal, clinical research, clinical trials, deep learning, machine learning, research design

## Abstract

**Background:**

Large language models (LLMs) are becoming increasingly popular in clinical trial design but have been underused in research proposal development.

**Objective:**

This study compared the performance of commonly used open access LLMs versus human proposal composition and review.

**Methods:**

A total of 10 LLMs were prompted to write a research proposal. Six physicians and each of the LLMs assessed 11 blinded proposals for capabilities and limitations in accuracy and comprehensiveness.

**Results:**

ChatGPT-o1 and Llama 3.1 were rated the most and least accurate, respectively, by human scorers. LLM scorers rated ChatGPT-o1 and DeepSeek R1 as the most accurate. ChatGPT-o1 and Llama 3.1 were rated as the most and least comprehensive, respectively, by human and LLM scorers. LLMs performed poorly on scoring proposals and, on average, rated proposals 1.9 points higher than humans for both accuracy and comprehensiveness.

**Conclusions:**

Paid versions of ChatGPT remain the highest-quality and most versatile option of the available LLMs. These tools cannot replace expert input but serve as powerful assistants, streamlining the development process and enhancing productivity.

## Introduction

Advancements in artificial intelligence (AI) have led to the development of large language models (LLMs) using algorithms that learn from data and recognize patterns to make decisions based on all available data within a training set [1]. However, AI is limited by the data it is trained on and an inability to account for the nuanced contexts of individual research studies [2]. Researchers are increasingly using LLMs in clinical trial design to improve patient selection, cohort composition, and recruitment [3]. In contrast, the use of LLMs in research proposal development is largely unexplored, and thus, they are perhaps underused. This study aimed to address this gap by comparing the performance of LLMs versus the current gold standard of human proposal composition and review. Our goals were 3-fold: to rate LLMs in composing clinical trial proposals, assess LLMs in scoring clinical trial proposals, and evaluate the ease of using LLMs (including usability and efficiency).

## Methods

### Overview

Commonly used open access AI platforms (DeepSeek R1, ChatGPT-o3-mini [OpenAI], ChatGPT-o1 [OpenAI], ChatGPT-4o [OpenAI], Claude Sonnet [Anthropic], Claude Opus [Anthropic], OpenEvidence, Grok 2 [xAI], Gemini Advanced [Google], and Llama 3.1 [Meta AI]) were evaluated for use in research proposal drafting. We requested each of the models to do the following:

Write a research proposal for a study looking at the use of narrowband-ultraviolet B phototherapy for psoriasis treatment for psoriasis patients of varying skin pigmentation with 3 aims: 1. To understand the factors that affect the response of NB-UVB in psoriasis patients of varying skin pigmentation. 2. Evaluate adverse effects of NB-UVB and their impact on psoriasis patients of varying skin pigmentation. 3. Compare the acute immunologic response to NB-UVB in psoriasis patients of varying skin pigmentation using bulk and single-cell RNA sequencing. Include the following sections: 1 page ‘Specific Aims’ with details on each of the 3 aims, 1/2 page background and significance of the topic, 1 page of ‘preliminary data/studies’ relevant to the study, 1 page ‘experimental design’ (include summary of study, inclusion and exclusion criteria, study visits and procedures with an associated table describing specifics of study visits), 1/2 page of ‘statistical methods, power calculations and bioinformatic analyses’ specific for each aim, 1/4 page of ‘potential problems and alternative strategies.’ Please have approximately 30 references from reputable sources. Make the proposal a total of 7 pages long in paragraph form, in formal scientific language and at a graduate level.

To assess the outputs, each of the 11 blinded proposals (n=10, 90.9% LLM generated and n=1, 9.1% human written) was systematically reviewed and scored by 6 independent physician evaluators, all with strong research backgrounds. Each evaluator used a standardized Likert scale ranging from 1 to 5 (1=“strongly disagree”; 5=“strongly agree”) to rate each proposal for capabilities and limitations in the LLMs’ accuracy and comprehensiveness (Table 1).

**Table 1.** Criteria for assessing the accuracy, usability, comprehensiveness, and efficiency of large language models (LLMs).

Domain	Assessment criteria	Scoring methodology
Accuracy	Raters systematically fact-checked all proposal content. Only proposals with fully correct and verified factual information (including cited data, statistics, and conclusions) were rated highly. All references were checked for verifiability, relevance, and reputable source quality.	Rated independently by each evaluator on a Likert scale from 1 to 5 (1=“strongly disagree: not accurate”; 5=“strongly agree: fully accurate”). Scores were aggregated by calculating the mean of all raters’ scores for each proposal.
Comprehensiveness	Assessed by evaluating inclusion and completeness of required proposal sections: specific aims, background and significance, preliminary data and studies, experimental design with inclusion and exclusion criteria and study visits and procedures, statistical methods, power calculations and bioinformatic analyses, and potential problems and alternative strategies. Proposals were further checked to meet format requirements: approximately 7 pages in length and 30 reputable references.	Rated independently on a Likert scale from 1 to 5. The mean score was calculated for all evaluators per proposal.
Usability	Assessed qualitatively based on researchers’ (MH and DC) experience using each LLM. Criteria included intuitiveness of the interface, clarity of documentation, and ease of generating proposals without technical guidance.	Rated by 2 nontechnical investigators on a Likert scale from 1 to 5; scores were descriptively summarized.
Efficiency	The time from user input to final output was measured in minutes. Minimal delays and rapid response were rated favorably.	The time (minutes and seconds) for the LLM to complete the query was recorded.

For each domain assessed by human reviewers, individual scores were first tabulated. Scores from the 6 evaluators for each proposal were then aggregated by calculating the mean domain score, yielding an overall mean score per domain for each proposal. These aggregated scores provided a quantitative measure of each proposal’s performance relative to evaluator consensus. No additional weighting was applied; each evaluator’s score carried equal weight in the final aggregation.

In addition to scientific content review, LLM usability and efficiency, including description of pros and cons, were evaluated by 2 investigators. These qualitative evaluations were collected separately and did not contribute to the aggregated proposal scores.

### Ethical Considerations

The authors have adhered to local, national, regional, and international law and regulations regarding protection of personal information, privacy, and human rights. This study did not involve human participants, identifiable private information, or interactions requiring human subjects protections. Accordingly, formal human ethics review approval was not required, and informed consent was not necessary. All data used in this study were deidentified prior to analysis to ensure participant confidentiality. No compensation was provided for participation in this study. These determinations are in accordance with University of Michigan policies and federal regulations (45 CFR 46) governing human research [4]. The research was conducted in compliance with the University of Michigan’s guidelines on research ethics.

## Results

### LLMs Composing Proposals

The human-written proposal obtained a score of 5 for accuracy and comprehensiveness across all human scorers and remained the gold standard (Table 2). Human scorers rated ChatGPT-o1 as the most accurate and Llama 3.1 as the least accurate. When assessed in scoring LLM-derived clinical trial proposals, LLM scorers rated ChatGPT-o1 and DeepSeek R1 as the most accurate (Multimedia Appendix 1). ChatGPT-o1 and Llama 3.1 were found to be the most and least comprehensive, respectively, by both human and LLM scorers.

**Table 2.** Full scores by evaluation criterion for each proposal and model.

Proposal and model	Accuracy (1-5), mean (SD)	Comprehensiveness (1-5), mean (SD)	Usability (1-5), mean (SD)	Efficiency
ChatGPT-4o	2.2 (1.2)	1.8 (1.4)	5.0 (0.0)	1 min, 37 s
Claude Opus	3.3 (1.4)	2.7 (0.6)	5.0 (0.0)	1 min, 30 s
ChatGPT-o1	3.5 (1.6)	4.3 (0.5)	3.5 (0.7)	1 min
ChatGPT-o3-mini	2.8 (1.7)	4.0 (0.6)	4.0 (0.0)	30 s
Claude Sonnet	2.0 (1.3)	1.8 (0.8)	4.0 (0.0)	28 s
DeepSeek R1	3.2 (1.5)	3.3 (1.4)	4.0 (0.0)	1 min, 23 s
OpenEvidence	2.3 (1.5)	1.3 (0.5)	3.5 (0.7)	45 s
Grok 2	3.2 (1.5)	3.0 (0.6)	4.0 (0.0)	1 min, 15 s
Gemini Advanced	2.5 (1.0)	1.5 (0.5)	4.5 (0.7)	37 s
Llama 3.1	1.7 (1.0)	1.5 (0.8)	4.5 (0.7)	20 s
Human proposal	5.0 (0.0)	5.0 (0.0)	N/A^a^	N/A (>10 working d)

a N/A: not applicable.

Mean and SD scores per criterion are reported for each proposal and model as assessed by 6 independent physician raters (except for usability, which was rated by 2 nontechnical investigators). Efficiency is reported as actual proposal generation time.

All raw scores are available in Multimedia Appendix 1.

### LLMs Scoring Proposals

Overall, LLMs performed poorly on scoring proposals and, on average, rated proposals 1.9 points higher than humans for both accuracy (range 1.3-2.8) and comprehensiveness (range 0.7-3). The Claude Sonnet proposal showed the largest discrepancy between human and LLM scoring, with an average difference of 2.8 (SD 3.4) points for accuracy and 3 (SD 4.2) points for comprehensiveness. Interestingly, the ChatGPT-o1 and DeepSeek proposals both received top scores of 5 for both accuracy and comprehensiveness from all LLMs versus human averages of 4.3 (SD 2.2) and 3.3 (SD 1.9), respectively. The absence of variance at the top of the range (and wide variance in the middle of the range) suggests that the discriminatory power of the LLMs plateaued at the top LLM quality.

### Ease of Using LLMs

All open access LLMs were highly efficient and ran in a matter of seconds to minutes (minimum of 20 seconds for Llama 3.1 and maximum of 1 minute and 37 seconds for ChatGPT-4o). When assessed for ease of use, ChatGPT-4o and Claude Opus offered the most intuitive interfaces and were highly usable for researchers (DC and MH) without computer science backgrounds.

## Discussion

### Principal Findings

LLMs offer powerful tools to assist humans in clinical trial proposal creation. LLMs take only minutes to generate proposals, whereas prior investigations into time commitment for generation of proposals by humans have reported estimates of 116 principal investigator hours, 55 coinvestigator hours, and 38 working days [5,6]. Therefore, judicious use of LLMs in proposal development allows researchers to save significant time in organizing sections, formatting, and ensuring coherence.

To provide guidance for readers, we performed a direct comparison of the tested LLMs, highlighting meaningful differences in performance, usability, and application. Table 3 summarizes these findings, with clear delineation of unique strengths and limitations for each model.

**Table 3.** Pros and cons of open access large language models (LLMs).

LLM (AI platform)	Pros	Cons
Overall	Generally reliable, very user-friendly, and highly comprehensive and efficient	Occasional factual inaccuracies and hallucinations (eg, fabricated references) Lack of access to the most recent studies due to their training data cutoffs^a^
ChatGPT	Most advanced and versatile option of the available LLMs GPT-4o is the lowest-latency^b^ and cheapest model	Offers more advanced, paid “reasoning” models (GPT-o1 and GPT-o3), but they are computationally expensive and slower
Claude	Designed with emphasis on alignment with human values Tends to be more cautious about controversial or sensitive topics	Models less tailored to clinical contexts compared to ChatGPT
DeepSeek	Fully open source, promoting transparency and community contributions Does not have associated license fees	Struggles with fine-tuning on dialogue Large models (eg, DeepSeek-Coder-33B) require large amounts of GPU^c^ memory
Gemini	Gemini 1.5 Pro boasts the largest context window^d^ as a part of Google’s ecosystem Gemini 1.5 Flash is one of the fastest models	Struggles to produce quality responses without significant prompt engineering Concerns about data privacy and use with integration into various Google services
Grok 2	Integration into X’s (formerly known as Twitter) ecosystem allows Grok to stay up-to-date with current events and trends Offers conversational capabilities tailored for social interaction	Remains suboptimal compared to Claude 3.5 or GPT-4o As a result of being directly linked to X, a platform with frequent user-generated content, Grok struggles to moderate sensitive or controversial interactions
Llama 3.1	Llama 3.2 is one of the fastest models (along with Gemini 1.5) Optimized for efficiency with lower computational requirements compared to other models	Technical expertise required for it to run properly Less user-friendly for researchers without technical support
OpenEvidence	Offers access to the most recently curated medical research Most robust and relevant citations	Weaker reasoning capabilities than those of leading frontier models

a LLM training data cutoffs: October 2023 for ChatGPT, April 2024 for Claude Sonnet and July 2024 for Claude Haiku, December 2023 for Llama 3.1, May 2024 for Gemini, and unknown for OpenEvidence and Grok.

b Time to first token of tokens received, in seconds, after the application programming interface request is sent.

c GPU: graphics processing unit.

d Maximum number of combined input and output tokens.

ChatGPT-o1 and ChatGPT-o3-mini demonstrated the highest overall accuracy and comprehensiveness, delivering well-structured proposals with robust citations and high scientific rigor. Llama 3.1 and Gemini Advanced were notably efficient, reliably delivering full proposals with rapid turnaround times, but occasionally produced less nuanced sections in preliminary data or limited discussion. Regarding ease of use, ChatGPT-4o and Claude Opus feature intuitive interfaces and require minimal learning curves, making them ideal for researchers new to AI-powered tools. In contrast, Llama 3.1 and OpenEvidence ranked the lowest in usability as their technical requirements and specialized interfaces can be challenging for new users.

All open access LLMs can aid in initial outlining and creation of research proposals. They can assist in initial brainstorming of a clear researchable question and generating hypotheses based on existing literature. LLMs are useful in literature review and can summarize existing studies related to the proposal topic and identify gaps in current knowledge. Furthermore, all open access LLMs can propose data collection methods, define eligibility criteria based on study objectives, recommend appropriate statistical tests based on study design, and help draft proposal sections. They also allow for iterative refinements, enabling tailored outputs to meet specific requirements or needs. While human verification is always required, LLMs can greatly improve time spent on initial proposal drafting and aid in mundane tasks associated with proposal writing, including proofreading and revisions, writing administrative sections, and optimizing citations.

### Limitations to Consider

All LLMs operate similarly to traditional autocomplete and work by using available contextual clues and a statistical model to predict the most likely next “token” or word. Due to the training data cutoffs of AI models, researchers must manually incorporate the latest literature findings. AI researchers are working on incorporating more access to real-time data, for example, generative pretrained transformer actions [6], but these solutions come with their own trade-offs. Another limitation is that users must verify citations as the model may “hallucinate” or fabricate realistic-sounding but false information. Finally, although AI models such as DALL-E (or others) can create images, they are less effective at producing accurate, clinical-grade figures.

Additionally, current LLMs were largely unable to score proposals and should not replace human review for quality control. The high scores from the LLM raters indicate that the LLMs were unable to detect entire missed protocol sections. Other than Gemini Advanced (who self-scored its written proposal with 3 for accuracy and comprehensiveness), Claude Sonnet, and Llama 3.1, all the LLMs self-scored their own proposals with 5 for both accuracy and comprehensiveness, suggesting overlapping “blind spots” in LLM proposal generation and evaluation.

One limitation of this study is that the order in which the proposals were sent for respondents to review was not randomized. Additionally, the “gold standard” (human proposal) was last, and question order likely played a role, with kinder grading of the LLM-derived proposals before reviewing the human-written proposal. Had the human proposal been first, this would have highlighted missing components of LLM-derived proposals and likely led to harsher human grading of the latter.

Another important limitation is the rapid and frequent versioning of LLM platforms, which poses challenges for scientific reproducibility. As models are updated, their performance and outputs can meaningfully change over time, making it difficult to reproduce results or maintain consistency in studies that rely on AI-generated content. Researchers should document model versions and use dates to mitigate this issue and ensure transparency.

### Conclusions

The future of AI in clinical research is expected to be transformative and far-reaching. As AI algorithms continue to evolve, they are likely to become more accurate, comprehensive, efficient, and interpretable, enabling researchers to leverage AI-driven insights for personalized medicine, disease prevention, and improved patient outcomes. In the coming years, AI is anticipated to play a crucial role in optimizing clinical trial design and accelerating drug discovery [7]. The integration of AI with other emerging technologies, such as blockchain and the Internet of Medical Things, could further revolutionize clinical research by improving data security, privacy, and real-time patient monitoring [8]. As these advancements continue to unfold, AI has the potential to democratize access to novel therapies, reduce health care costs, and, ultimately, usher in an era of precision medicine [9].

LLMs offer a transformative approach to drafting research proposals [10]. Paid versions of ChatGPT (ChatGPT-o3-mini and ChatGPT-o1) currently remain the highest-quality (as determined by the Artificial Analysis Quality Index) and most versatile option of the available LLMs, balancing usability, speed, accuracy, and customization [11]. While these tools cannot entirely replace expert input, they serve as powerful assistants, streamlining the development process and enhancing productivity. For optimal results, researchers should combine AI-generated content with their expertise, ensuring precision and adherence to the latest research standards.

## Supplementary material

10.2196/76674 Multimedia Appendix 1 Human and large language model (LLM) scoring of LLM performance on accuracy and comprehensiveness.

## Abbreviations

AI: artificial intelligence
LLM: large language model
