# Supplementary material for: Leveraging AI Large Language Models for Writing Clinical Trial Proposals in Dermatology: Instrument Validation Study
Source: JMIR Dermatol. 2026 Jan 12;9:e76674. doi: 10.2196/76674 (PMC12795409; doi:10.2196/76674)
Supplement: Multimedia Appendix 1 [file derma-v9-e76674-s001.docx]

Supplementary Table S1. Human and LLM scoring LLMs on accuracy and comprehensiveness

|  | **Human Scoring** | | | | | | | **AI Scoring** | | | | | | | | | |  |  |
| --- | --- | --- | --- | --- | --- | --- | --- | --- | --- | --- | --- | --- | --- | --- | --- | --- | --- | --- | --- |
|  | **1** | **2** | **3** | **4** | **5** | **6** | **Average (Var)** | **Deepseek R1** | **ChatGPT o3-mini** | **ChatGPT o1** | **ChatGPT 4o** | **Claude Sonnet** | **Claude Opus** | **Grok 2** | **Gemini Advanced** | **Llama 3.1** | **Average (Var)** | **Overall average** |  |
| **Accuracy** | | | | | | | | | | | | | | | | | | | |
| Human Proposal (Control) | 5 | 5 | 5 | 5 | 5 | 5 | **5 (0)** | 5 | 5 | 5 | 5 | 5 | 5 | 5 | 4 | 5 | **4.9 (0.1)** | **4.9** |  |
| ChatGPT o1 | 1 | 4 | 2 | 5 | 5 | 4 | **3.5 (2.7)** | 5 | 5 | 5 | 5 | 5 | 5 | 5 | 5 | 5 | **5 (0)** | **4.4** |  |
| Deepseek R1 | 2 | 4 | 1 | 4 | 5 | 3 | **3.2 (2.2)** | 5 | 5 | 5 | 5 | 5 | 5 | 5 | 5 | 5 | **5 (0)** | **4.3** |  |
| Claude Opus | 3 | 4 | 1 | 3 | 4 | 5 | **3.3 (1.9)** | 5 | 5 | 5 | 5 | 5 | 5 | 5 | 4 | 5 | **4.9 (0.1)** | **4.3** |  |
| ChatGPT o3-mini | 1 | 4 | 1 | 4 | 5 | 2 | **2.8 (3)** | 5 | 5 | 5 | 5 | 5 | 5 | 5 | 4 | 5 | **4.9 (0.1)** | **4.1** |  |
| Grok 2 | 2 | 4 | 1 | 3 | 5 | 3 | **3.2 (2)** | 5 | 5 | 5 | 5 | 5 | 4 | 5 | 4 | 5 | **4.8 (0.2)** | **4.1** |  |
| Claude Sonnet | 1 | 1 | 1 | 4 | 3 | 2 | **2 (1.6)** | 5 | 5 | 5 | 5 | 4 | 4 | 5 | 5 | 5 | **4.8 (0.2)** | **3.7** |  |
| OpenEvidence | 1 | 1 | 5 | 2 | 3 | 2 | **2.3 (2.3)** | 5 | 5 | 4 | 5 | 4 | 4 | 5 | 5 | 5 | **4.7 (0.3)** | **3.7** |  |
| Gemini Advanced | 2 | 4 | 1 | 3 | 3 | 2 | **2.5 (1.1)** | 5 | 4 | 5 | 5 | 3 | 4 | 5 | 3 | 5 | **4.3 (0.8)** | **3.6** |  |
| ChatGPT 4o | 1 | 2 | 1 | 4 | 3 | 2 | **2.2 (1.4)** | 5 | 5 | 5 | 5 | 3 | 3 | 4 | 4 | 5 | **4.3 (0.8)** | **3.5** |  |
| Llama 3.1 | 1 | 3 | 1 | 3 | 1 | 1 | **1.7 (1.1)** | 3 | 2 | 4 | 5 | 2 | 2 | 3 | 2 | 4 | **3 (1.3)** | **2.5** |  |
| **Comprehensiveness** | | | | | | | | | | | | | | | | | | | |
| Human Proposal (Control) | 5 | 5 | 5 | 5 | 5 | 5 | **5 (0)** | 5 | 4 | 5 | 5 | 5 | 5 | 5 | 4 | 5 | **4.8 (0.2)** | **4.9** |  |
| ChatGPT o1 | 4 | 4 | 4 | 5 | 5 | 4 | **4.3 (0.3)** | 5 | 5 | 5 | 5 | 5 | 5 | 5 | 5 | 5 | **5 (0)** | **4.7** |  |
| ChatGPT o3-mini | 4 | 4 | 3 | 4 | 5 | 4 | **4 (0.4)** | 5 | 5 | 5 | 5 | 5 | 5 | 5 | 4 | 5 | **4.9 (0.1)** | **4.5** |  |
| Deepseek R1 | 4 | 4 | 1 | 3 | 5 | 3 | **3.3 (1.9)** | 5 | 5 | 5 | 5 | 5 | 5 | 5 | 5 | 5 | **5 (0)** | **4.3** |  |
| Claude Opus | 4 | 3 | 1 | 3 | 1 | 4 | **2.7 (1.9)** | 5 | 5 | 5 | 5 | 5 | 5 | 5 | 4 | 5 | **4.9 (0.1)** | **4** |  |
| Grok 2 | 3 | 3 | 4 | 3 | 3 | 2 | **3 (0.4)** | 5 | 5 | 5 | 5 | 5 | 4 | 5 | 3 | 5 | **4.7 (0.5)** | **4** |  |
| Claude Sonnet | 2 | 1 | 2 | 3 | 1 | 2 | **1.8 (0.6)** | 5 | 5 | 5 | 5 | 5 | 3 | 5 | 5 | 5 | **4.8 (0.4)** | **3.6** |  |
| ChatGPT 4o | 2 | 2 | 1 | 2 | 2 | 2 | **1.8 (0.2)** | 5 | 5 | 5 | 5 | 3 | 3 | 4 | 4 | 5 | **4.3 (0.8)** | **3.3** |  |
| OpenEvidence | 2 | 1 | 2 | 1 | 1 | 1 | **1.3 (0.3)** | 4 | 5 | 2 | 5 | 3 | 2 | 3 | 3 | 5 | **3.6 (1.5)** | **2.7** |  |
| Gemini Advanced | 1 | 1 | 2 | 2 | 1 | 2 | **1.5 (0.3)** | 4 | 2 | 3 | 5 | 3 | 3 | 4 | 3 | 5 | **3.56 (1)** | **2.7** |  |
| Llama 3.1 | 1 | 1 | 1 | 3 | 1 | 2 | **1.5 (0.7)** | 3 | 2 | 3 | 5 | 2 | 1 | 3 | 2 | 5 | **2.9 (1.9)** | **2.3** |  |
